# Green tea dietary supplementation in broiler chickens: Effect on the development of chicken intestine

**DOI:** 10.1002/fsn3.2126

**Published:** 2021-01-19

**Authors:** Wuyi Liu, Fariba Rouzmehr, Xin Wang, Alireza Seidavi

**Affiliations:** ^1^ Department of Biological Sciences Anhui engineering technology research center of Anti‐aging Chinese Herbal Medicine Fuyang Normal University Fuyang City China; ^2^ Department of Animal Science Rasht Branch Islamic Azad University Rasht Iran

**Keywords:** dietary effect, feed additives, green tea, intestine, supplement

## Abstract

This experiment explored the dietary effects of green tea (*Camellia sinensis*) in feed supplementation on the development of broiler chicks. Totally, two hundred and seventy‐day‐old male broiler chicks were assigned to 27 broiler groups each with 10 individuals (initial mean body weight 44.2 ± 1.3 g) in a 4 × 2 factorial arrangement. Each chicken group was supplemented with the feed additives of green tea powder. The trial data were measured and obtained based on the records of carcass traits and intestine characteristics of broiler chicken fed with four different additive levels of green tea (0.25%, 0.50%, 0.75%, and 1.00%). The experiment lasted for two trial periods of 21 days and 42 days for each treatment of the green tea supplement with full records of broiler traits. There were interesting results recorded in the majority of broiler intestinal traits between the two trial periods. There are a few significant differences (*p* < .05) observed among multiple comparisons of some intestinal traits in broiler chicks such as colon diameter (*p* = .022) and jejunum width (*p* = .01). The most significant differences exist in these intestinal traits of chicken right and left cecum among broiler chicks fed with dietary green tea powder (*p* < .05). The other intestinal characteristics of broiler chicks were recorded from single treatment are insignificantly distinguished compared with the control groups. There are also some near significant differences of chicken intestinal carcass traits and characteristics. These results and experimental data of this study extend the current knowledge on the dietary effects of green tea in chicken raising and feeding with dietary supplementation.

## Graphical abstract

This experiment explored the dietary effects of green tea (Camellia sinensis) in feed supplementation on the development of broiler chicks. Significant statistics of broiler chicken intestine traits of colon diameter and jejunum width were affected by different additive levels of green tea at the 42nd day of age. The trial results suggest that there are some significant improvements in two traits, i.e. colon diameter (overall *p* = .022) and jejunum width (overall *p* = .01) among broiler chicks fed with green tea.

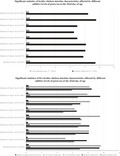

## 1 INTRODUCTION

In the daily life, consumers focused more and more on poultry feed and production and carcass composition, with especial interests in those natural dietary ingredients of domestic animals. With the widespread application of agricultural internet of things, the public often discuss about the hot issues of food and commodity safety, such as the natural extractives and biochemical derivatives from plants perceived by consumers. Green tea (*Camellia sinensis*) is widely analyzed and used in biomedicine and physiology for its positive responses as feed additives and natural feed supplementations and alternative ingredients of antibiotics (Cao et al., 2005; Kojima & Yoshida, 2008). Green tea is regarded as potential valuable sources of good nutrients and antibiotic replacement and safe natural antioxidants for both human and animals (Cyril & Jozef, 2017; Hamer, 2007; Khan & Mukhtar, 2007; Liao et al., 2001). Green tea has been famous for its remarkable bioactive extractive or derivatives utilized as effective medicinal components in both ancient and modern medicines for several centuries (Hamer, 2007; Wu & Wei, 2002). Therefore, green tea and its extractives or derivatives are widely utilized as natural feed ingredients and important additives for feeding domestic animals (Cao et al., 2005; Ishihara, 2001; Jelveh et al., 2018; Seidavi et al., 2017; Suzuki et al., 2002; Yang et al., 2003). Moreover, positive bioactivities and dietary effects of green tea feed additives on performance have been frequently observed and reported in domestic animals (Jelveh et al., 2018; Saraee et al., 2014, 2015; Seidavi et al., 2014, 2017). However, there are rare experiments (e.g., Chen et al., 2019; Hassanpour et al., 2010; Kaya et al., 2018) that fully reported the effect of feeding green tea additives or its biochemical derivatives on broiler intestine. To our knowledge, there are only seven feeding experiments reported on the dietary effects of green tea supplementations or other dietary acidic feed additives on the development of chicken intestine (Chen et al., 2019; Emamgholi Begli et al., 2017; Hassanpour et al., 2010; Hosseini et al., 2017; Kaya et al., 2018; Lemos et al., 2015; Tzora et al., 2017). Compared with the control group, the duodenum villous length and jejunum villous length and their surface areas were observed and reported as significantly developed (*p* < .05) in the green tea‐treated groups (Hassanpour et al., 2010). Recently, Chen et al. (2019) examine and evaluate the effects of green tea and mulberry leaf powders on chicken gut microflora and animal health too. However, at present, there are only a few trials and/or cases that reported the pharmacological effects of dietary tea on animal gastrointestinal or intestinal epithelial cells (Acharyya et al., 2015; Koo & Cho, 2004; Pranjkovíc & Šola, 2013; Song et al., 2011; Vermaak et al., 2009; Yang et al., 2001).

In recent years, the issues of the physiological maintenance and improvement of intestinal morphology are major hotspots in gut health topics for both human and animal nutritional studies. It is generally recognized that gut health is far more complex than just on modulation of the gut microflora through sole probiotics. Due to a more stabilized livestock intestinal health, animals are less exposed to biological toxins and toxic microbiota or microbial metabolites with other undesired environmental risk factors. Therefore, it is necessary to conduct studies on the issues of intestinal development and its physiological effects on gut health. In addition, we hypothesized that green tea has a positive effect on intestinal morphology and development of broiler chickens because of its antioxidant effects in organism. The experiment was designed to explore and analyze the dietary effects of green tea in feed supplementation on the development of broiler chicks during the raising phases. It was to make good use of green tea and its extractive or derivatives in chicken and other domestic animals.

## 2 MATERIALS AND METHODS

### 2.1 Animals and feeding

The experiment was done in a 4 × 2 factorial arrangement with four dietary green tea powder levels (0.25%, 0.50%, 0.75%, and 1.00%) and two durations of waste green tea powder (21 and 42 days). Two hundred and seventy one‐day‐old male broiler chicks were allocated to twenty‐seven groups, and each group was assembled with ten birds of Ross 308 broiler strain purchased from Aviagen Ltd UK (Tompic et al., 2011; please see the webpage of Aviagen Ltd in Newbridge and Scotland in UK for details). The initial mean body weight of each trial group was computed as 44.2 ± 1.3 g. The broiler chickens were put and dealt in the similar feeding programs and housing dimensions of cages as those reported in the previous trials (Liu et al., 2018; Seidavi et al., 2017). In Tables 1 and 2, it was showed that the combined feed ingredient and dietary composition for these trial chicks raised in the experiment. The diets was made and developed on the corresponding feed values of Ross 308 chicken catalog recommendations, whereas the housing feed and water were offered in voluntary intakes for these trial chicks. The trial was conducted with the parameters and treatments of temperature, humidity, light, ventilation, and feeding of housing and feeding following the previous trials (Liu et al., 2018; Seidavi et al., 2017). However, it was carried out with a control treatment and eight treatments of four dietary levels (0.25%, 0.50%, 0.75%, and 1.00%) of green tea powder in this experiment.

**TABLE 1:** Feed ingredients of used diets during the starter (1st–21st days of age) and finisher (22nd–42nd days of age) periods

Treatment ingredient (%)	Starter (1st−21st days of age)					Finisher (22nd−42nd days of age)				
Green tea powder	0	2.5	5.0	7.5	10.0	0	2.5	5.0	7.5	10.0
Corn	557.0	555.5	553.9	552.3	550.8	598.0	596.1	594.7	593.3	591.7
Soybean meal	370.9	369.8	368.9	368.0	366.9	323.3	322.5	321.4	320.1	319.2
Soybean oil	28.2	28.2	28.2	28.2	28.2	40.2	40.4	40.3	40.3	40.3
Di‐calcium phosphate	19.4	19.4	19.4	19.4	19.4	17.0	17.0	17.0	17.0	17.0
Carbonate calcium	13.5	13.5	13.5	13.5	13.5	11.3	11.3	11.3	11.3	11.3
Vitamin mixture^a^	2.5	2.5	2.5	2.5	2.5	2.5	2.5	2.5	2.5	2.5
Mineral mixture^b^	2.5	2.5	2.5	2.5	2.5	2.5	2.5	2.5	2.5	2.5
NaCl	1.9	1.9	1.9	1.9	1.9	2.0	2.0	2.0	2.0	2.0
Bicarbonate sodium	2.4	2.4	2.4	2.4	2.4	2.2	0.22	2.2	2.2	2.2
DL‐Methionine	1.3	1.3	1.3	1.3	1.3	1.0	1.0	1.0	1.1	1.1
L‐Lysine‐Hydro‐Chloride	0.4	0.5	0.5	0.5	0.6	0	0	0.1	0.2	0.2
Total	1,000	1,000	1,000	1,000	1,000	1,000	1,000	1,000	1,000	1,000
Price (Rial/kg)	15,564	15,784	15,998	16,213	16,432	15,254	15,474	15,691	15,936	16,150

^a^ Vitamin A: 1.65 mg/g (i.e., 5,000 IU/g); Vitamin D3: 0.0125 mg/g (i.e., 500 IU/g); Vitamin E: 3 mg/g (i.e., 3 IU/g); Vitamin K3: 1.5 mg/g; Vitamin B2: 1 mg/g

^b^ Calcium Pantothenate: 4 mg/g; Niacin: 15 mg/g; Vitamin B6: 13 mg/g; Cu: 3 mg/g; Zn: 15 mg/g; Mn: 20 mg/g; Fe: 10 mg/g; K: 0.3 mg/g

**TABLE 2:** Nutrient analysis of used diets during the starter (1st–21st days of age) and finisher (22nd–42nd days of age) periods

Treatment ingredient (%)	Starter (1st−21st days of age)					Finisher (22nd−42nd days of age)				
Dry Matter (%)	86.644	86.646	86.657	86.668	86.671	87.159	87.172	87.173	87.176	87.188
Metabolizable energy (kcal/kg)	3.025	3.025	3.025	3.025	3.025	3.150	3.150	3.150	3.150	3.150
Crud Protein (%)	23.003	23.003	23.003	23.002	23.003	21.000	21.002	21.003	21.000	21.000
Ether Extract (%)	5.308	5.305	5.302	5.298	5.296	6.616	6.632	6.619	6.616	6.613
Linoleic Acid (%)	2.812	2.808	2.804	2.800	2.797	3.495	3.501	3.492	3.489	3.485
Crude fiber (%)	2.672	2.704	2.737	2.770	2.803	2.576	2.609	2.642	2.674	2.707
Calcium (%)	1.052	1.051	1.051	1.051	1.050	0.903	0.903	0.903	0.902	0.902
Total Phosphorus (%)	0.745	0.744	0.743	0.742	0.741	0.682	0.681	0.680	0.679	0.678
Available Phosphorus (%)	0.504	0.503	0.503	0.502	0.502	0.452	0.452	0.451	0.451	0.450
Potassium (%)	0.919	0.916	0.914	0.912	0.909	0.835	0.833	0.830	0.827	0.825
Chloride (%)	0.163	0.165	0.165	0.164	0.166	0.160	0.160	0.162	0.164	0.164
Manganese (mg/kg)	403.604	403.549	403.502	403.456	403.401	400.382	400.338	400.284	400.221	400.174
Sodium (mg/kg)	0.163	0.163	0.162	0.162	0.162	0.160	0.160	0.160	0.160	0.160
Zinc (mg/kg)	324.201	324.136	324.080	324.023	323.959	322.229	322.174	322.110	322.038	321.981
Choline (g/kg)	1.578	1.574	1.571	1.567	1.563	1.474	1.470	1.466	1.462	1.459
Folic acid (mg/kg)	2.108	2.103	2.100	2.096	2.091	1.953	1.949	1.945	1.940	1.936
Arginine (%)	1.502	1.498	1.494	1.491	1.486	1.352	1.349	1.344	1.339	1.336
Glycine (%)	0.944	0.941	0.939	0.937	0.934	0.860	0.858	0.855	0.852	0.850
Serine (%)	1.126	1.123	1.120	1.117	1.114	1.023	1.020	1.017	1.013	1.011
Glycine + Serine (%)	2.441	2.434	2.428	2.422	2.415	2.206	2.201	2.194	2.185	2.179
Histidine (%)	0.603	0.601	0.600	0.598	0.596	0.551	0.550	0.548	0.546	0.545
Iso‐Leucine (%)	0.948	0.945	0.943	0.940	0.938	0.859	0.857	0.854	0.851	0.848
Leucine (%)	1.944	1.939	1.934	1.929	1.923	1.807	1.802	1.797	1.790	1.786
Lysine (%)	1.272	1.276	1.273	1.270	1.274	1.112	1.110	1.113	1.117	1.114
Methionine (%)	0.476	0.475	0.474	0.473	0.472	0.422	0.421	0.420	0.426	0.425
Cysteine (%)	0.367	0.366	0.365	0.364	0.363	0.340	0.339	0.338	0.337	0.336
Methionine + Cysteine (%)	0.843	0.841	0.840	0.838	0.836	0.763	0.761	0.759	0.766	0.765
Phenylalanine (%)	1.080	1.076	1.074	1.071	1.068	0.984	0.981	0.978	0.974	0.972
Tyrosine (%)	0.890	0.888	0.886	0.883	0.881	0.810	0.808	0.805	0.802	0.800
Phenylalanine + Tyrosine (%)	1.970	1.964	1.959	1.954	1.949	1.794	1.789	1.783	1.777	1.772
Threonine (%)	0.855	0.853	0.850	0.848	0.846	0.778	0.776	0.773	0.771	0.768
Tryptophan (%)	0.308	0.307	0.306	0.305	0.305	0.275	0.274	0.274	0.272	0.272
Valine (%)	1.046	1.043	1.041	1.038	1.035	0.957	0.954	0.951	0.948	0.945

### 2.2 Carcass dissection and measurements of intestinal morphology

At the 42nd day of age, after 4 hr of fasting, at least three chicken of each group (more than three chicken were initially selected for each group) having the nearest weight to the average weight (mean body weight 44.2 ± 1.3 g) of the herd were selected for the experiment. Afterward, the chicken neck, wingtips, gut, and liver were removed, whereas the empty and/or the edible carcass were weighed. Finally, the dimensions of intestinal segments were recorded. The total weight of all dissected parts and the weights of various segments of the digestive tract were related to the totally eviscerated carcass. The matching measurement ratios of chicken intestinal morphology were calculated according to the following formula ([weight of component/eviscerated carcass weight] × 100).

After 12 hr, they were slaughtered, and as 2 cm samples mainly from the three gut segments of the intestine were taken. In brief, these three gut segments are the middle part of the duodenum from the gizzard outlet to the end of the pancreatic loop, the middle part of the jejunum from the pancreatic loop to Meckel's diverticulum, and the 5 cm part from Meckel's diverticulum to the ileocaecocolic junction. After washing with soluble phosphate‐buffered saline (PBS), the samples were then transmitted into plastic containers containing 6 ml 10% formalin. The intestine segments one cm long were taken from the center of each part and fixed in 10% buffered formalin for morphometric studies under light microscopy. The intestine morphology of each part was numerically estimated and evaluated. Morphometric analysis was calculated and evaluated according to the previous reported methods of Iji et al. (2001) and Giannenas et al. (2011).

### 2.3 Statistical analysis

Experimental data were analyzed by analysis of variance based on a 4 × 2 factorial arrangement with two feeding durations (21 and 42 days) and four feeding levels of dietary green tea (0.25%, 0.50%, 0.75%, and 1.00% in diet).

The concrete calculation process was executed with SPSS package version 16.0 for Windows (SPSS Inc, C I, 1997) using a two‐way ANOVA procedure. All the data were also subjected to statistical analysis using General Linear Model procedures and Duncan's multiple range tests of the SPSS package Windows (SPSS Inc, C I, 1997).

## 3 RESULTS

The experimental data were obtained based on the practicable measures and observations of carcass traits and characteristics of chicken intestine. All the trial results and dealt data were summarized in Tables 3, 4, 5, 6, 7, 8 for Ross 308 broilers. These intestinal characteristics are analyzed as follows.

**TABLE 3:** Statistical mean (±*SEM*) of cranial gut segments at the 42nd day of age in Ross 308 broilers affected by four different additive levels of green tea and two different durations of its usage*

Treatment	Trait	Crop weight (g)	Relative weight of crop (%)	Proventriculus weight (g)	Relative weight of proventriculus (%)	Pancreas weight (g)	Relative weight of pancreas (%)
Use duration (day)	21	7.377^a^	0.269^a^	11.909^a^	0.432^a^	6.079^a^	0.221^a^
	42	7.528^a^	0.283^a^	12.307^a^	0.460^a^	5.552^a^	0.208^a^
*p*		.718	.441	.796	.595	.164	.306
*SEM* (Standard Error of Mean)		0.290	0.013	1.073	0.037	0.256	0.009
Green tea powder level (%)	0.25	6.563^b^	0.246^b^	11.903^a^	0.444^a^	5.503^a^	0.205^a^
	0.50	7.657^ab^	0.277^ab^	12.602^a^	0.458^a^	6.017^a^	0.217^a^
	0.75	7.532^ab^	0.273^ab^	11.900^a^	0.430^a^	6.158^a^	0.221^a^
	1.00	8.057^a^	0.309^a^	12.028^a^	0.452^a^	5.583^a^	0.213^a^
*p*		.107	.153	.985	.983	.518	.807
*SEM* (Standard Error of Mean)		0.410	0.018	1.517	0.053	0.362	0.012
Duration (0 days)**‐** level (0%)		7.667^ab^	0.267^ab^	10.583^a^	0.371^a^	6.183^ab^	0.219^a^
Duration (21 days)**‐** level (0.25%)		6.903^ab^	0.265^ab^	12.240^a^	0.467^a^	5.847^ab^	0.224^a^
Duration (42 days)**‐** level (0.25%)		6.223^b^	0.227^b^	11.567^a^	0.421^a^	5.160^b^	0.186^a^
Duration (21 days)**‐** level (0.50%)		8.010^ab^	0.280^ab^	10.527^a^	0.365^a^	6.150^ab^	0.214^a^
Duration (42 days)**‐** level (0.50%)		7.303^ab^	0.274^ab^	14.677^a^	0.550^a^	5.883^ab^	0.220^a^
Duration (21 days)**‐** level (0.75%)		6.843^ab^	0.246^b^	10.827^a^	0.386^a^	6.917^a^	0.246^a^
Duration (42 days)**‐** level (0.75%)		8.220^a^	0.300^ab^	12.973^a^	0.473^a^	5.400^ab^	0.197^a^
Duration (21 days)**‐** level (1.00%)		7.750^ab^	0.286^ab^	14.043^a^	0.508^a^	5.403^ab^	0.198^a^
Duration (42 days)**‐** level (1.00%)		8.363^a^	0.331^a^	10.013^a^	0.397^a^	5.763^ab^	0.228^a^
*p*		.226	.217	.711	.571	.402	.487
*SEM* (Standard Error of Mean)		0.583	0.024	2.026	0.070	0.501	0.019

* Means (±standard error of means) within each column of dietary treatments with no common superscript differ significantly at *p* < .05.

**TABLE 4:** Statistical mean (±*SEM*) of duodenum characteristics at the 42nd day of age in Ross 308 broilers affected by four different additive levels of green tea and two different durations of its usage*

Treatment	Trait	Duodenum weight (g)	Relative weight of duodenum (%)	Duodenum length (mm)	Duodenum width (mm)	Duodenum diameter (mm)
Use duration (day)	21	21.296^a^	0.774^a^	39.457^a^	6.182^a^	0.318^a^
	42	20.577^a^	0.769^a^	33.327^a^	6.277^a^	0.367^a^
*p*		.555	.887	.118	.849	.354
*SEM* (Standard Error of Mean)		0.843	0.026	2.621	0.348	0.036
Green tea powder level (%)	0.25	20.267^a^	0.751^a^	37.898^a^	6.687^a^	0.320^ab^
	0.50	22.053^a^	0.801^a^	34.500^a^	6.067^a^	0.410^a^
	0.75	19.732^a^	0.710^a^	32.178^a^	5.777^a^	0.240^b^
	1.00	21.695^a^	0.824^a^	40.992^a^	6.387^a^	0.398^ab^
*p*		.476	.159	.384	.599	.111
*SEM* (Standard Error of Mean)		1.192	0.036	3.707	0.492	0.051
Duration (0 days)**‐** level (0%)		19.930^a^	0.705^ab^	33.333^ab^	6.597^a^	0.373^a^
Duration (21 days)**‐** level (0.25%)		21.550^a^	0.821^ab^	42.797^ab^	5.857^a^	0.270^a^
Duration (42 days)**‐** level (0.25%)		18.983^a^	0.681^b^	33.000^ab^	7.517^a^	0.370^a^
Duration (21 days)**‐** level (0.50%)		20.597^a^	0.720^ab^	33.333^ab^	5.900^a^	0.347^a^
Duration (42 days)**‐** level (0.50%)		23.510^a^	0.882^a^	35.667^ab^	6.233^a^	0.473^a^
Duration (21 days)**‐** level (0.75%)		19.907^a^	0.709^ab^	33.140^ab^	6.257^a^	0.243^a^
Duration (42 days)**‐** level (0.75%)		19.557^a^	0.711^ab^	31.217^b^	5.297^a^	0.237^a^
Duration (21 days)**‐** level (1.00%)		23.130^a^	0.846^ab^	48.557^a^	6.713^a^	0.410^a^
Duration (42 days)**‐** level (1.00%)		20.260^a^	0.802^ab^	33.427^ab^	6.060^a^	0.387^a^
*p*		.521	.171	.281	.634	.361
*SEM* (Standard Error of Mean)		1.649	0.057	4.944	0.716	0.074

* Means (±standard error of means) within each column of dietary treatments with no common superscript differ significantly at *p* < .05.

**TABLE 5:** Statistical mean (±*SEM*) of jejunum characteristics at the 42nd day of age in Ross 308 broilers affected by four different additive levels of green tea and two different durations of its usage*

Treatment	Trait	Jejunum weight (g)	Relative weight of jejunum (%)	Jejunum length (mm)	Jejunum width (mm)	Jejunum diameter (mm)
Use duration (day)	21	48.484^a^	1.758^a^	60.918^a^	8.013^a^	0.403^a^
	42	46.688^a^	1.750^a^	63.497^a^	7.628^a^	0.393^a^
*p*		.643	.954	.455	.347	.863
*SEM* (Standard Error of Mean)		2.686	0.093	2.382	0.281	0.040
Green tea powder level (%)	0.25	42.592^a^	1.585^a^	62.818^ab^	7.946^ab^	0.380^a^
	0.50	52.027^a^	1.884^a^	69.333^a^	7.577^b^	0.392^a^
	0.75	46.250^a^	1.662^a^	53.772^b^	6.757^b^	0.350^a^
	1.00	49.477^a^	1.884^a^	62.907^ab^	9.003^a^	0.468^a^
*p*		.357	.290	.037	.009	.525
*SEM* (Standard Error of Mean)		3.799	0.132	3.369	0.398	0.057
Duration (0 days)**‐** level (0%)		52.847^a^	1.837^a^	69.333^a^	9.457^a^	0.377^a^
Duration (21 days)**‐** level (0.25%)		44.227^a^	1.692^a^	61.970^ab^	7.813^abc^	0.357^a^
Duration (42 days)**‐** level (0.25%)		40.957^a^	1.479^a^	63.667^a^	8.080^abc^	0.403^a^
Duration (21 days)**‐** level (0.50%)		49.850^a^	1.740^a^	67.000^a^	8.160^abc^	0.413^a^
Duration (42 days)**‐** level (0.50%)		54.203^a^	2.027^a^	71.667^a^	6.993^bc^	0.370^a^
Duration (21 days)**‐** level (0.75%)		48.073^a^	1.704^a^	47.897^b^	6.527^c^	0.370^a^
Duration (42 days)**‐** level (0.75%)		44.427^a^	1.619^a^	59.647^ab^	6.987^bc^	0.330^a^
Duration (21 days)**‐** level (1.00%)		51.787^a^	1.895^a^	66.807^a^	9.553^a^	0.470^a^
Duration (42 days)**‐** level (1.00%)		47.167^a^	1.874^a^	59.007^ab^	8.453^ab^	0.467^a^
*p*		.744	.630	.063	.010	.922
*SEM* (Standard Error of Mean)		5.585	0.186	4.623	0.554	0.079

* Means (± standard error of means) within each column of dietary treatments with no common superscript differ significantly at *p* < .05.

**TABLE 6:** Statistical mean (±*SEM*) of ileum characteristics at the 42nd day of age in Ross 308 broilers affected by four different additive levels of green tea and two different durations of its usage*

Treatment	Trait	Ileum weight (g)	Relative weight of ileum (%)	Ileum length (mm)	Ileum width (mm)	Ileum diameter (mm)
Use duration (day)	21	47.251^a^	1.715^a^	70.514^a^	7.052^a^	0.353^a^
	42	47.583^a^	1.777^a^	70.018^a^	7.470^a^	0.347^a^
*p*		.947	.732	.902	.425	.749
*SEM* (Standard Error of Mean)		3.503	0.127	2.796	0.361	0.045
Green tea powder level (%)	0.25	43.993^a^	1.632^a^	68.620^ab^	7.090^a^	0.307^a^
	0.50	55.992^a^	2.027^a^	76.167^a^	6.785^a^	0.465^a^
	0.75	45.125^a^	1.620^a^	63.135^b^	6.963^a^	0.305^a^
	1.00	44.558^a^	1.705^a^	73.142^ab^	8.205^a^	0.378^a^
*p*		.296	.368	.146	.233	.279
*SEM* (Standard Error of Mean)		4.954	0.180	3.955	0.510	0.064
Duration (0 days)**‐** level (0%)		50.680^a^	1.769^a^	77.667^a^	7.837^ab^	0.323^ab^
Duration (21 days)**‐** level (0.25%)		44.187^a^	1.692^a^	66.573^a^	6.227^ab^	0.300^ab^
Duration (42 days)**‐** level (0.25%)		43.800^a^	1.572^a^	70.667^a^	7.953^ab^	0.313^ab^
Duration (21 days)**‐** level (0.50%)		56.083^a^	1.959^a^	76.667^a^	5.970^b^	0.347^ab^
Duration (42 days)**‐** level (0.50%)		55.900^a^	2.095^a^	75.667^a^	7.600^ab^	0.583^a^
Duration (21 days)**‐** level (0.75%)		43.410^a^	1.540^a^	61.423^a^	7.523^ab^	0.360^ab^
Duration (42 days)**‐** level (0.75%)		46.840^a^	1.700^a^	64.847^a^	6.403^ab^	0.250^b^
Duration (21 days)**‐** level (1.00%)		45.323^a^	1.668^a^	77.393^a^	8.487^a^	0.407^ab^
Duration (42 days)**‐** level (1.00%)		43.793^a^	1.742^a^	68.890^a^	7.923^ab^	0.350^ab^
*p*		.763	.807	.304	.162	.346
*SEM* (Standard Error of Mean)		6.653	0.240	5.306	0.681	0.086

* Means (±standard error of means) within each column of dietary treatments with no common superscript differ significantly at *p* < .05.

**TABLE 7:** Statistical mean (±*SEM*) of colon characteristics at the 42nd day of age in Ross 308 broilers affected by four different additive levels of green tea and two different durations of its usage*

Treatment	Trait	Colon weight (g)	Relative weight of colon (%)	Colon length (mm)	Colon width (mm)	Colon diameter (mm)
Use duration (day)	21	4.817^a^	0.174^b^	7.534^a^	8.191^a^	0.421^a^
	42	6.129^a^	0.228^a^	7.168^a^	8.404^a^	0.400^a^
*p*		.093	.046	.350	.670	.524
*SEM* (Standard Error of Mean)		0.519	0.018	0.268	0.348	0.023
Green tea powder level (%)	0.25	4.922^a^	0.181^a^	7.463^ab^	8.082^a^	0.358^a^
	0.50	5.995^a^	0.218^a^	8.083^a^	7.960^a^	0.457^a^
	0.75	6.180^a^	0.221^a^	6.463^b^	8.348^a^	0.380^a^
	1.00	4.797^a^	0.183^a^	7.390^ab^	8.800^a^	0.447^a^
*p*		.441	.528	.056	.641	.115
*SEM* (Standard Error of Mean)		0.734	0.025	0.380	0.492	0.032
Duration (0 days)**‐** level (0%)		5.183^ab^	0.179^ab^	7.667^ab^	9.163^a^	0.537^a^
Duration (21 days)**‐** level (0.25%)		3.613^b^	0.137^b^	7.770^ab^	7.080^a^	0.300^d^
Duration (42 days)**‐** level (0.25%)		6.230^ab^	0.224^ab^	7.167^ab^	9.083^a^	0.417^abcd^
Duration (21 days)**‐** level (0.50%)		4.660^ab^	0.162^ab^	7.833^ab^	7.257^a^	0.450^abc^
Duration (42 days)**‐** level (0.50%)		7.330^a^	0.274^a^	8.833^a^	8.663^a^	0.463^abc^
Duration (21 days)**‐** level (0.75%)		6.173^ab^	0.219^ab^	6.677^ab^	9.300^a^	0.427^abcd^
Duration (42 days)**‐** level (0.75%)		6.187^ab^	0.223^ab^	6.250^b^	7.397^a^	0.333^cd^
Duration (21 days)**‐** level (1.00%)		4.823^ab^	0.177^ab^	7.857^ab^	9.127^a^	0.507^ab^
Duration (42 days)**‐** level (1.00%)		4.770^ab^	0.189^ab^	6.923^ab^	8.473^a^	0.387^bcd^
*p*		.381	.273	.172	.181	.022
*SEM* (Standard Error of Mean)		1.051	0.035	0.518	0.704	0.043

* Means (±standard error of means) within each column of dietary treatments with no common superscript differ significantly at *p* < .05.

**TABLE 8:** Statistical mean (±*SEM*) of right and left cecum characteristics at the 42nd day of age in Ross 308 broilers affected by four different additive levels of green tea and two different durations of its usage*

Treatment	Trait	Right cecum weight (g)	Relative weight of right cecum (%)	Right cecum length (mm)	Right cecum width (mm)	Right cecum diameter (mm)	Left cecum weight (g)	Relative weight of left cecum (%)	Left cecum length (mm)	Left cecum width (mm)	Left cecum diameter (mm)
Use duration (day)	21	7.457^a^	0.270^a^	16.829^a^	7.751^b^	0.295^a^	6.573^b^	0.238^b^	17.192^a^	8.127^b^	0.284^a^
	42	8.767^a^	0.329^a^	17.568^a^	10.207^a^	0.252^a^	8.513^a^	0.319^a^	17.520^a^	10.725^a^	0.253^a^
*p*		.174	.109	.387	.022	.160	.007	.004	.720	.017	.217
*SEM* (Standard Error of Mean)		0.652	0.025	0.588	0.685	0.020	0.446	0.017	0.634	0.688	0.017
Green tea powder level (%)	0.25	6.980^a^	0.259^a^	15.855^b^	8.988^a^	0.268^a^	6.585^a^	0.244^a^	16.522^a^	7.950^a^	0.290^a^
	0.50	9.702^a^	0.352^a^	18.583^a^	9.890^a^	0.277^a^	8.288^a^	0.301^a^	18.167^a^	10.328^a^	0.260^a^
	0.75	7.842^a^	0.283^a^	16.837^ab^	8.528^a^	0.265^a^	7.257^a^	0.262^a^	17.300^a^	8.973^a^	0.240^a^
	1.00	7.923^a^	0.303^a^	17.530^ab^	8.508^a^	0.285^a^	8.042^a^	0.308^a^	17.437^a^	10.452^a^	0.283^a^
*p*		.243	.317	.168	.724	.961	.251	.238	.646	.253	.482
*SEM* (Standard Error of Mean)		0.922	0.035	0.831	0.969	0.029	0.631	0.024	0.897	0.973	0.025
Duration (0 days)**‐** level (0%)		8.990^ab^	0.308^ab^	18.000^ab^	10.190^ab^	0.347^a^	9.637^a^	0.334^ab^	18.167^a^	10.217^ab^	0.313^ab^
Duration (21 days)**‐** level (0.25%)		4.697^b^	0.180^b^	14.877^b^	5.520^b^	0.283^ab^	5.067^b^	0.194^c^	16.210^a^	5.420^b^	0.343^a^
Duration (42 days)**‐** level (0.25%)		9.263^ab^	0.339^ab^	16.833^ab^	12.457^a^	0.253^ab^	8.103^ab^	0.295^abc^	16.833^a^	10.480^a^	0.237^ab^
Duration (21 days)**‐** level (0.50%)		8.103^ab^	0.282^ab^	17.167^ab^	9.527^ab^	0.273^ab^	7.047^ab^	0.245^abc^	17.167^a^	8.387^ab^	0.223^b^
Duration (42 days)**‐** level (0.50%)		11.300^a^	0.422^a^	20.000^a^	10.253^ab^	0.280^ab^	9.530^a^	0.357^a^	19.167^a^	12.270^a^	0.297^ab^
Duration (21 days)**‐** level (0.75%)		8.617^ab^	0.308^ab^	16.573^ab^	8.447^ab^	0.327^ab^	6.270^ab^	0.223^bc^	17.193^a^	8.757^ab^	0.297^ab^
Duration (42 days)**‐** level (0.75%)		7.067^ab^	0.258^ab^	17.080^ab^	8.610^ab^	0.203^b^	8.243^ab^	0.301^abc^	17.407^a^	12.270^ab^	0.213^b^
Duration (21 days)**‐** level (1.00%)		8.410^ab^	0.310^ab^	18.700^ab^	7.510^b^	0.297^ab^	7.907^ab^	0.290^abc^	18.200^a^	8.757^ab^	0.303^ab^
Duration (42 days)**‐** level (1.00%)		7.437^ab^	0.296^ab^	16.360^ab^	9.507^ab^	0.273^ab^	8.177^ab^	0.325^ab^	16.673^a^	9.190^a^	0.263^ab^
*p*		.281	.257	.189	.151	.387	.097	.076	.795	.145	.201
*SEM* (Standard Error of Mean)		1.542	0.054	1.157	1.461	0.039	1.022	0.036	1.220	1.446	0.035

* Means (±standard error of means) within each column of dietary treatments with no common superscript differ significantly at *p* < .05.

In Table 3, the statistics show the development of the cranial gut segments of broiler chicken raised at the 42nd day of age fed with four different additive levels of green tea (0.25%, 0.50%, 0.75%, and 1.00%). These trial results suggest there are significant improvements (*p* < .05) in two traits of chicken crop or craw (i.e., the absolute and relative weights of chicken crop) among broiler chicks fed with green tea, especially the extreme cases of 0.25% and 1.00%. No significant results are observed in the other four traits among broiler chicks (Table 3), such as the absolute and relative weights of chicken proventriculus. There are no significant results recorded in all the six traits among broiler chicks between the use duration groups of 21 days and 42 days either. However, there are a few significant differences observed in the two trait measures of absolute and relative weights of chicken crop among broiler chicks, when pair‐wise comparisons are considered and counted between the feed additive use durations of 21 and 42 days (Table 3). For instance, these of 0.25%, 0.75%, and 1.00% green tea feeding levels of 42 days versus those of the other green tea feeding levels of 42 days and 21 days and the control groups (Table 3). The results reveal an efficient individual growth and organ development in chicken initial digestive organ (i.e., the crop mucosa) fed with four levels of green tea.

In Table 4, the statistics reveal the development of the duodenum characteristics of broiler chicken raised at the 42nd day of age affected by four different additive levels of green tea (0.25%, 0.50%, 0.75%, and 1.00%). These trial results suggest that there are many improvements of chicken intestinal duodenum characteristics but only overall significant improvements (*p* < .05) were found in the duodenum diameter among broiler chicks treated with different supplementary levels of green tea, especially the middle treatment levels of 0.50% and 0.75%. However, no significant results are observed among specific pair‐wise comparisons of different green tea feeding levels of these five traits among broiler chicks in consideration of the feed additive use durations of 21 and 42 days (Table 4). There are many insignificant results recorded in all the five broiler traits between the use durations of 21 and 42 days, such as the absolute and relative weights of chicken duodenum. Meanwhile, there are a few significant differences observed in the trait of broiler chicken duodenum length when specific pair‐wise comparisons of different trial groups are considered and dealt, especially between those two treated groups of 0.75% and 1.00% (Table 4). The results reveal an efficient growth and organ development in chicken duodenum fed with green tea.

In Table 5, the statistics indicate the development of the jejunum characteristics of broiler chicken raised at the 42nd day of age much affected by four different additive levels of green tea (0.25%, 0.50%, 0.75%, and 1.00%). These trial results suggest that there are some significant improvements among multiple comparisons of two traits, that is, jejunum length (*p* = .063) and jejunum width (*p* = .01) in broiler chicks fed with green tea (Table 5). No significant results are observed in the other three traits (Table 5). There are no significant results recorded in all the five broiler traits between the use durations of 21 and 42 days either (Table 5). However, there are a few significant pair‐wise differences (*p* < .05) observed in some traits, including jejunum length and jejunum width, among broiler chicks when specific pair‐wise comparisons of different trial groups are considered and dealt (Table 5). The results reveal an efficient growth and organ development in chicken jejunum fed with four levels of green tea.

In Table 6, the statistics exhibit the development of the ileum characteristics of broiler chicken raised at the 42nd day of age affected by four different additive levels of green tea (0.25%, 0.50%, 0.75%, and 1.00%). The results suggest that there are some significant pair‐wise improvements (*p* < .05) only in one trait (i.e., ileum length) among broiler chicks fed with four levels of green tea, but no significant results are observed in the other four traits among broiler chicks (Table 6). There are no significant results recorded in all the five broiler traits among broiler chicks between the use durations of 21 days and 42 days either. However, there are a few significant pair‐wise differences (*p* < .05) observed in two traits (i.e., ileum width and ileum diameter) among broiler chicks, when specific pair‐wise comparisons of different trial groups are considered and dealt, especially between the two treated groups of 0.50% and 0.75% (Table 6).

In Table 7, the statistics display the development of the colon characteristics of broiler chicken raised at the 42nd day of age affected by four different additive levels of green tea (0.25%, 0.50%, 0.75%, and 1.00%). The results suggest that there are significant improvements (*p* < .05) merely in the trait of colon diameter among broiler chicks fed with four levels of green tea. However, no significant results are observed in the other four traits among broiler chicks (Table 7). There is only one significant observation in the relative weight of colon among broiler chicks between the use durations of 21 and 42 days (Table 7). Meanwhile, there are also a few significant differences (*p* < .05) observed in four traits, that is, the absolute weight of colon, the relative weight of colon, colon length, and colon diameter, among broiler chicks, when specific pair‐wise comparisons of the treatment and control groups are considered and dealt (Table 7). These results reveal an efficient growth and organ development in chicken colon fed with four levels of green tea.

In Table 8, the statistics uncover the development of the right and left cecum characteristics of broiler chicken raised at the 42nd day of age affected by four different additive levels of green tea (0.25%, 0.50%, 0.75%, and 1.00%). The results suggest that there are some significant pair‐wise improvements (*p* < .05) in the trait of right cecum length among broiler chicks fed with green tea. However, no significant results are observed in the other seven traits among broiler chicks (Table 8). There are three significant observations in three chicken traits (i.e., the right and left cecum weight and the relative weight of left cecum) among the treated broiler chicks. There are also a few significant pair‐wise differences (*p* < .05) observed in all these traits except for left cecum length among broiler chicks, when specific pair‐wise comparisons of different trial groups are considered and dealt (Table 8). These results reveal an efficient growth and organ development in chicken right and left cecum fed with green tea.

### 3.1 Discussion

In summary, among multiple comparisons of all the broiler traits, there are a few significant differences (*p* < .05) observed in the absolute and relative weights of chicken crop (Table 3). These results reveal an efficient individual growth and organ development in chicken initial digestive organ (i.e., the crop mucosa) fed with four levels of green tea (Table 3). These results agree well with the work reported by Janssens et al. (2016) that reported green tea supplementation for 12 weeks did not have a significant effect on composition of the human gut segments and gut microbiota.

Furthermore, there are also many insignificant results recorded in all the five broiler traits between the use durations of 21 and 42 days (Table 4), such as the absolute and relative weights of chicken duodenum. There are a few significant pair‐wise differences (*p* < .05) observed in the trait of broiler chicken duodenum length when specific pair‐wise comparisons of different trial groups are considered, especially between those two treated groups of 0.75% and 1.00% (Table 4). Those results reveal an efficient growth and organ development in chicken duodenum fed with green tea. These results agree well with the previous work reported by Hassanpour et al. (2010) who reported that the duodenum villous length and surface area of the green tea supplemented groups were significantly improved compared with the control group. They found no significant differences in the chicken duodenum villous width of green tea supplemented groups either (Hassanpour et al., 2010), which agrees well with our observed results.

Moreover, the trial results suggest that there are some significant or near significant improvements among multiple comparisons of two traits, jejunum length (*p* = .063) and jejunum width (*p* = .01), in broiler chicks fed with green tea (Table 5). At the same time, there are also a few significant pair‐wise differences (*p* < .05) observed in these traits besides of jejunum length and jejunum width among broiler chicks when specific pair‐wise comparisons of different trial groups are considered and dealt (Table 5). The results reveal an efficient growth and organ development in chicken jejunum fed with four levels of green tea. These results agree well with the experiment reported by Hassanpour et al. (2010). They observed that the length and surface area of the chicken jejunum villi in the green tea‐supplemented groups significantly increased compared with the control group (Hassanpour et al., 2010), while the width of jejunum villi was significantly increased in the control group (*p* < .05) too (Hassanpour et al., 2010).

The trial results suggest that there are some significant improvements (*p* < .05) in single trait (i.e., ileum length) among broiler chicks fed with green tea, but no significant results are observed in the other four traits among broiler chicks (Table 6). There are no significant results recorded in all the five broiler traits among broiler chicks between the use durations of 21 and 42 days either. However, there are a few significant pair‐wise differences (*p* < .05) observed in two traits (i.e., ileum width and ileum diameter) among broiler chicks, when specific pair‐wise comparisons of different trial groups are considered and dealt, especially between those two treated groups of 0.50% and 0.75% (Table 6). These results reveal an efficient growth and organ development in chicken ileum fed with four levels of green tea. These results also agree partly with the experiment reported by Hassanpour et al. (2010) who discovered that the ileum villous length, width, surface area, lamina propria, and muscle layer thickness had not significantly changed. However, our observed results suggested the ileum villous length was obviously improved (*p* < .05) fed with different additive levels of green tea.

In the meantime, there are a few significant pair‐wise differences (*p* < .05) observed in four traits, especially colon diameter (Table 7). For example, these traits among broiler chicks like the absolute weight of colon, the relative weight of colon, the colon length, and the colon diameter, when specific pair‐wise comparisons of different trial groups are considered and dealt, especially those pair‐wise results in the chicken trait of colon diameter (Table 7). These results reveal an efficient growth and organ development in chicken colon fed with green tea.

Meanwhile, the trial results suggest that there are significant pair‐wise improvements (*p* < .05) only in the trait of right cecum length among broiler chicks fed with four levels of green tea (Table 8). There are also three significant observations in three chicken traits (i.e., the right and left cecum weight and the relative weight of left cecum) among the treated broiler chicks (Table 8). There are also a few significant pair‐wise differences (*p* < .05) observed in all these traits except for left cecum length among broiler chicks, when specific pair‐wise comparisons of different trial groups are considered and dealt (Table 8). These results reveal an efficient growth and organ development in chicken right and left cecum fed with four levels of green tea.

As shown in Tables 7, 8, these statistics and observed results agree well with the previous work reported by Hosseini et al. (2017) who found that Thymolina® powder decreased microbial activity in the terminal ileum, cecum, and colon, which would affect the development of these gut segments. On the whole, most of the significant differences of intestinal characteristics among broiler chicks fed with dietary green tea exist in these intestinal traits of right and left cecum of chicken, compared with the control groups (Tables 3, 4, 5, 6, 7, 8). However, there are the obvious decreasing and increasing trends of some measured developmental characteristics of chicken intestine (i.e., the weights of crop, the measurements of duodenum diameter, duodenum length, jejunum length, and jejunum width) observed as impacted by the feed supplementation of green tea. In brief, the intestinal statistics of characteristics from the records of single treatment are insignificant. However, there are also some near significant pair‐wise differences (*p* < .05) of duodenum diameter (mm), crop weight (g), and relative weight of crop (%) found in the treatments of green tea at the 42nd day of age (Tables 3, 4, 5, 6, 7, 8). Further, there are some significant differences of chicken jejunum width (mm) and colon diameter (mm). Moreover, near significant differences of chicken jejunum length (mm) were also detected at the significant pair‐wise differences of *p* < .05 in chicken intestine (Tables 5, 6, 7). These trial results agree well with the previous work (Hassanpour et al., 2010), in which a few significant results were reported in the parameters of developing intestines and many insignificant parameters of chicken intestinal morphology (Hassanpour et al., 2010). They found no significant differences among chicken duodenum villous width, jejunum villous width, lamina propria and muscle layer thickness, and ileum villous length and width and surface area (Hassanpour et al., 2010). Furthermore, they also reported no significant differences among those measured intestinal parameters at three intestinal parts (Hassanpour et al., 2010). The jejunum villous length and surface area significantly increased among all the green tea treated groups (Hassanpour et al., 2010). Meanwhile, the length and surface area of chicken duodenal villi of the 4% green tea treatment group were larger than those of the control group (*p* < .05), but the differences were reported as statistically significant (*p* < .05) too (Hassanpour et al., 2010).

In fact, some similar results to the experiment were also reported (Hosseini et al., 2017; Tzora et al., 2017). It was reported that Thymolina® powder could increase trypsin and amylase activity in the homogenate of pancreas and small intestine, and chime content in jejunum (Hosseini et al., 2017). However, Thymolina^®^ powder could reduce microbial activity in the terminal ileum, cecum, and colon (Hosseini et al., 2017). It was observed that a significant difference existed between dietary Thymolina^®^ treatments in broiler intestinal morphology parameters, including the statistics of chicken villus height, villus width, crypt depth, and epithelial thickness (Hosseini et al., 2017). In addition, it was found that the combined use of these feed additives increased E. jejuni counts and increased cell proliferation in the duodenum and jejunum (Tzora et al., 2017). Meanwhile, benzoic acid improved intestinal wall morphology in the ileum (Tzora et al., 2017). There are obvious decreasing and increasing trends of some measured characteristics of chicken intestine during the trial periods (Tables 3, 4, 5, 6, 7, 8). For instance, there are obvious decreasing trends of relative weight of crop (%), proventriculus weight (g), pancreas weight (g), duodenum width (mm), jejunum length (mm), colon length (mm), colon width (mm), colon diameter (mm), right cecum weight (g), left and right width (mm) of cecum, left diameter (mm) and right width (mm) of cecum, and relative weight of right cecum (%). Meanwhile, there are subtle increasing trends of relative weight (%) of pancreas and duodenum, width (mm) and diameter (mm) of jejunum, absolute weight (g), and relative weight (%) of ileum too.

Moreover, animals are generally seldom exposed to biological toxins and the noxious microbiota and microbial metabolites due to more stabilized livestock intestinal health in the farms. In addition, animal feed additives of growth promoting relieve the host animals from immune defense stress during the development phases and growth periods. Thus, the feed additives can be used to raise the intestinal availability with the essential nutrients for animal internal absorption and help animals to grow and develop better. The present experimental data contribute much to our understanding the dietary effects of green tea feed supplementation and bring insights into how to increase the animal well‐being and healthy development of chicken intestine.

## CONFLICT OF INTEREST

The authors report and declare no conflicts of any interest.

## AUTHOR CONTRIBUTIONS

See the authors' contribution declaration / statement.

## ETHIC STATEMENT OF STUDIES INVOLVING ANIMAL SUBJECTS

The study's protocols and procedures were ethically reviewed and approved by the Ethic Committee of Islamic Azad University. The chicken experimental cares were taken to minimize the number of trial animals used, and the study was conducted according to the International Guidelines for research involving animals (Directive 11750103931004, issued at 4 February 2015).

## CONSENT FOR PUBLICATION

The authors approved the consent for publication.

## Data Availability

Data and material are available for research purpose and for reference.
